# Molecular Confirmation, Epidemiology, and Pathophysiology of *Ehrlichia canis* Prevalence in Eastern India

**DOI:** 10.3390/pathogens13090803

**Published:** 2024-09-16

**Authors:** Ankita Chakraborty, Prasana Kumar Rath, Susen Kumar Panda, Bidyut Prava Mishra, Manaswini Dehuri, Sangram Biswal, Manoj Kumar Jena, Basanta Pravas Sahu, Biswaranjan Paital, Dipak Kumar Sahoo

**Affiliations:** 1 Department of Veterinary Pathology, College of Veterinary Science and Animal Husbandry, Odisha University of Agriculture and Technology, Bhubaneswar 751003, Odisha, India; 2 Department of Livestock Products Technology, College of Veterinary Science and Animal Husbandry, Odisha University of Agriculture and Technology, Bhubaneswar 751003, Odisha, India; 3 Department of Veterinary Parasitology, College of Veterinary Science and Animal Husbandry, Odisha University of Agriculture and Technology (OUAT), Bhubaneswar 751003, Odisha, India; 4 Department of Preventive Veterinary Medicine, College of Veterinary Science and Animal Husbandry, Odisha University of Agriculture and Technology (OUAT), Bhubaneswar 751003, Odisha, India; 5 Department of Biotechnology, School of Bioengineering and Biosciences, Lovely Professional University, Phagwara 144411, Punjab, India; 6 School of Biological Sciences, The University of Hong Kong, Pok Fu Lam, Hong Kong 999077, China; 7 Redox Regulation Laboratory, Department of Zoology, College of Basic Science and Humanities, Odisha University of Agriculture and Technology, Bhubaneswar 751003, Odisha, India; 8 Veterinary Clinical Sciences, College of Veterinary Medicine, Iowa State University, Ames, IA 50011, USA

**Keywords:** Canine Ehrlichiosis, dog’s pathophysiology, *Ehrlichia* epidemiology, molecular identification, p28 gene, ticks, ultrasonography

## Abstract

The present study aimed to investigate pathological epidemiology and molecular confirmation of *Ehrlichia canis* among pet dogs in Bhubaneswar, Odisha, a state in eastern India. A total of 178 dogs were screened for Ehrlichiosis based on history, clinical signs, blood, and buffy coat smear examination, resulting in only 56 dogs (31.46%) screening positive. The epidemiological study recorded a non-significant (*p* ≥ 0.05) increase in incidences among male dogs (68%), German Shepherds (25%), dogs more than 20 kg body weight (75%), in the summer months (55%), and dogs housed in pukka houses with exposure to the outside (59%). The majority of the infected dogs had a history of tick infestation (79%) at some point in their lives. Clinical signs showed non-typical manifestations like fever, lethargy, diarrhoea, epistaxis, hind limb edema, and corneal opacity. Haematological studies revealed anaemia and thrombocytopenia along with neutrophilia with relative lymphopenia and monocytosis. A decreasing trend was observed in the levels of total protein and albumin, with an increase in the levels of globulin, alanine aminotransferase, alanine aminotransferase, aspartate aminotransferase, blood urea nitrogen, and creatinine. The ultrasonography studies revealed hepatosplenomegaly along with hyper-echogenicity in various organs. Proteinuria and haematuria were consistent, along with the presence of bile salts in the urine of affected dogs. Molecular confirmation from n-type PCR data using Ehrlichia-specific primers targeting the p28 gene (843 bp) was done, and the identified gene sequences submitted to NCBI databases have accession numbers OQ383671-OQ383674 and OP886674-OP886677. Ticks collected from dogs were identified morphologically through microscopy and scanning electron microscopy as *Rhipicephalus sanguineus*.

## 1. Introduction

Diseases transmitted through ticks are now regarded as an emerging problem around the globe. One such important and commonly known cosmopolitan tick-borne rickettsia disease is Canine Ehrlichiosis, with the first official report coming from Algeria in 1935 [1]. This disease is prevalent in both tropical and sub-tropical climates because of the hot and humid conditions that favour its occurrence, and is also referred to as tropical canine pancytopenia. *Ehrlichia canis*, an obligate intracytoplasmic gram-negative pleomorphic rickettsia organism of the family Anaplasmataceae [2], is the primary etiological agent for Canine Ehrlichiosis. Transmission mainly occurs by *Riphicephalus sanguineus,* the brown dog tick [3]. Among all the *Ehrlichia* species, *E. canis* is studied primarily for its widespread prevalence in the canine population [4,5]. Most clinical signs in affected dogs were non-specific and overlapping, making clinical diagnosis difficult.

The animals having an acute phase show symptoms such as lethargy, fever, dullness, nasal bleeding, petechial haemorrhage, myalgia, bruising, and weight loss, along with conditions including hepatomegaly, splenomegaly, lymphadenopathy, ascites, nephritis, and other cardiorespiratory and haematological disorders [2,6]. The most common haematological findings in Canine Ehrlichiosis are anaemia, leukopenia, and thrombocytopenia [6]. During the chronic stage, pancytopenia becomes the most characteristic finding, probably due to the destruction and impairment of the bone marrow function [2]. Affected dogs show altered serum biochemical parameters predominantly indicative of hepatic [7] and renal dysfunction [8]. On ultrasonography, hyperechogenicity of the liver is observed in most of the positive cases, along with hepatomegaly [8]. Ehrlichiosis is a multi-systemic disease. Necropsy of the affected dogs revealed hepatomegaly, generalised lymphadenopathy, contracted kidney, and petechial to ecchymotic haemorrhages in visceral organs [9,10]. Diagnosis of Ehrlichiosis was most often made by demonstrating morulae within the cytoplasm of monocytes through blood smear examination, whose sensitivity and specificity depended upon loads of parasites in the blood [11]. Detection of intracytoplasmic *E. canis* morulae in monocytes is more accessible during the acute stage of infection but quite tricky in sub-acute and chronic stages [11]. Haemato-biochemical alterations and ultrasonographic changes can help support the clinical signs and assist in reaching a preliminary diagnosis [4,12]. Detection of DNA of *E. canis* through the polymerase chain reaction (PCR) from blood and tissue is regarded as the most sensitive and confirmatory diagnosis [13]. The existing literature on Canine Ehrlichiosis in Odisha is quite limited. Given the widespread and growing prevalence of Canine Monocytic Ehrlichiosis (CME), a multisystemic disease in dogs that poses a significant threat to canines globally, the hypothesis of this study was that the disease is also widespread among dogs in Bhubaneswar, India. Accordingly, the objective of this study was to assess various epidemiological risk factors, haemato-biochemical alterations, and pathological changes, along with molecular confirmation of *E. canis* in the dog population of Bhubaneswar. Since such a disease has multiple physiological consequences, the obtained results would be highly useful to establish the molecular profile of the *Ehrlichia* sp. present in the dog population of the eastern Indian city on one hand, and it would raise awareness among pet owners and veterinarians in the state on the other hand.

## 2. Materials and Methods

### 2.1. Sample Area and Ethical Approach

Between March 2019 and December 2022, pet dogs (*n* = 178) presented to the Veterinary Clinical Complex (VCC) of the Veterinary College of the State (OUAT), located at Bhubaneswar, Odisha (20.1863° N, 85.6223° E of India) were screened for Canine Ehrlichiosis. Blood samples were collected after obtaining due consent from the pet owners for clinical investigations and the samples were stored at –80 °C for further molecular confirmation through PCR (model ProFlex™ PCR System, Thermo Fisher Scientific, Waltham, MA, USA). Blood and buffy coat smears were prepared and stained with Giemsa protocol to observe morulae in leucocytes through direct microscopy for initial screening, along with the history of tick infestations and other clinical signs related to Ehrlichiosis. Although qPCR is more sensitive to detecting lower pathogen load, ligated adapters and tailed primers can be useful to sequence qPCR products without further amplification. Due to lacking a facility, we used endpoint PCR to sequence the amplicons. However, this could limit the Ec-positive samples because of the low sensitivity associated with endpoint PCR.

### 2.2. Epidemiological Risk Factors

A questionnaire was prepared to gather the details of the presented dogs from the pet owners. The questionnaire recorded details related to their ages, sex, breed, type of housing, etc., and they were later analysed statistically by applying the Chi-square test using SAS software version 9.3 (SAS Institute Inc., Cary, NC, USA) to find out the level of significant associations between each risk factor influencing the occurrence of Canine Ehrlichiosis.

### 2.3. Haemato-Biochemical Examinations

Blood collected from 178 dogs (Control-122, Affected-56) for a routine haematology and serum biochemical study, processed through the automatic haematological analyser (Model: Medonic M51, Spånga, Sweden) and by the automated serum biochemical analyser (Model: Turbochem-100, CPC, Awareness Technology Inc, i-track, Palm City, FL, USA) were present at the veterinary clinical complex (VCC) in the college. The data recorded for various haemato-biochemical parameters were analysed by using SAS software (Version: W32_7PRO, Bangalore, India) through a Student’s t-test to observe any differences between infected and control groups. A *p* ≤ 0.05 value was accepted as statistically significant.

### 2.4. Molecular Confirmation through PCR, Sequencing, and Phylogenetic Study

Molecular detection of *Ehrlichia canis* was performed by targeting the p28 gene using conventional PCR [14]. DNA was isolated from whole blood using a QIAamp DNA mini kit, (QIAGEN, Hilden, Germany) The *Ehrlichia* spp. was amplified in the thermal cycler using a reaction mixture containing specially designed primers to amplify the 843 bp fragment from the p28 gene. The primers used were ECp28-F (f) 5′-ATGAATTGCAAAAAAATTCTTATA-3′ and ECp28-R (r) 5′-TTAGAAGTTAAA TCTTCC TCC-3′. Temperature time Protocol in the Proflex PCR system (model ProFlex™ PCR System, Thermo Fisher Scientific, Waltham, MA, USA) is listed in Table 1. The product obtained from the PCR was taken as the representative sample for the *Ehrlichia canis* parasite and was sent for sequencing at ILS (Institute of Life Science) Bhubaneswar, India. Sequencing was performed using the Sanger method in an Automated DNA sequencer (ABI Model: 3730XL, SeqGen, Inc., Torrance, CA, USA) by HKP SCIENTIFIC, Bhubaneswar, Odisha, India. The Bio Edit programme (Version 7.2, https://bioedit.software.informer.com/, accessed on 13 March 2024) was used for trimming the sequences. The phylogenetic tree was constructed using the sequencing results. The analysis was performed through BLAST using the website www.ncbi.nlm.nih.gov/BLAST, accessed on 13 March 2024. The sequences were aligned with similar sequences from India and other countries retrieved from the NCBI database using MEGAX software (The Biodesign Institute, Tempe, AZ, USA) followed by a phylogenetic tree construction using the neighbor-joining method with a bootstrap value of 1000 [15]. 

### 2.5. Ultrasonography 

Two-dimensional B-mode ultrasonography was conducted among positively screened dogs (*n* = 56) using the ultrasonography (Model: LOGIQ F8, GE Health Care, Chicago, IL, USA) expert in the imaging unit located in the veterinary clinical complex, Odisha University of Agriculture and Technology, Bhubaneswar, India. One 8C curvilinear probe was used in most affected dogs with a frequency range of 3.0–10.0 MHz. Ultrasonographic images are primarily intended to observe any systemic alterations such as hepatomegaly, splenomegaly, and presence or absence of ascitic fluid, etc., in affected dogs, which will be helpful in correlating with other haemato-biochemical alterations.

### 2.6. Identification of Ticks through Scanning Electron Microscopy (SEM)

The ticks (*n* = 30) were collected by forceps without damaging mouth parts and appendages and were put in labeled sterile plastic vials. In some cases, an attempt was made at SEM (S-3400, Hitachi, Tokyo, Japan) in the Central Instrumentation Facility (CIF), Odisha University of Agriculture and Technology, Bhubaneswar, India for further identification. A 5.00 KV voltage and distance varying from 9.8 mm to 10.0 mm with magnification ranging from ×40 SE to ×250 SE were used during scanning in SEM. 

### 2.7. Pathology

Necropsy was conducted for three dogs that died during the study period to record the characteristic morbid changes. Demonstrative tissue samples were collected and fixed in 10% neutral formalin for further processing at the Department of Veterinary Pathology, College of Veterinary Science & Animal Husbandry, Odisha University of Agriculture and Technology, Bhubaneswar, India to observe histopathological changes. 

## 3. Results

**Figure 1:**
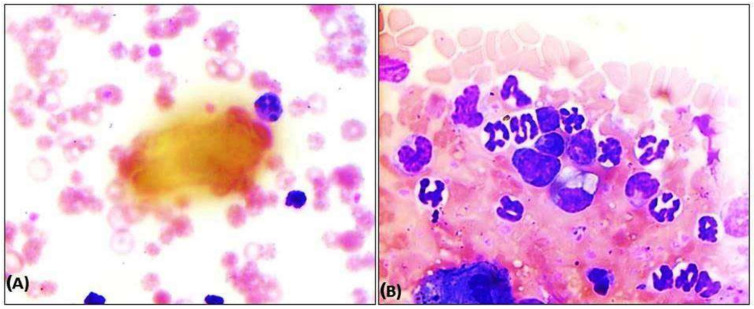
Occurrence of *Ehrlichia canis* in blood cells of buffy coat smear dogs in eastern India. (**A**) Presence of morula of *Ehrlichia canis* in neutrophils in blood smear, (**B**) Presence of morula of *Ehrlichia canis* in monocytes in buffy coat smear. The pictures taken with 100× magnification.

Out of the total dogs (*n* = 178) screened, 56 were found positive either through peripheral blood and buffy coat examinations or molecular confirmation through PCR, giving a prevalence rate of 31.46%. The other 122 dogs screened negative through examination of peripheral blood and buffy coat smear, and they were treated as control and apparently healthy dogs who were presented for routine vaccination, deworming etc. Three ehrlichiosis-positive dogs succumbed even after due treatment, giving a mortality rate of 1.68% and a case fatality rate of 5.35%. Preliminary screening through direct microscopy by observing the presence of any dark blue compact inclusions or morulae in leucocytes of the Giemsa-stained peripheral blood smears and buffy coat smears recorded an overall prevalence of 3.93% (*n* = 7) and 10.11% (*n* = 18), respectively. Most of the morula stages were prominent in monocytes (Figure 1A); however, they were also present in the cytoplasm of lymphocytes and neutrophils (Figure 1B), as observed in the present study. 

### 3.1. Epidemiological Risk Factors 

Details of epidemiological risk factors and their association with the occurrence of Canine Ehrlichiosis are listed in Table 2. Statistical analysis through the Chi-square test showed a non-significant (*p* ≥ 0.05) association for the breed, sex, housing pattern, body weight, and season in incidences of Ehrlichiosis in dogs (Table 2). However, the occurrence of Canine Ehrlichiosis significantly varied among different age groups of dogs. 

### 3.2. Clinical Signs

**Figure 2:**
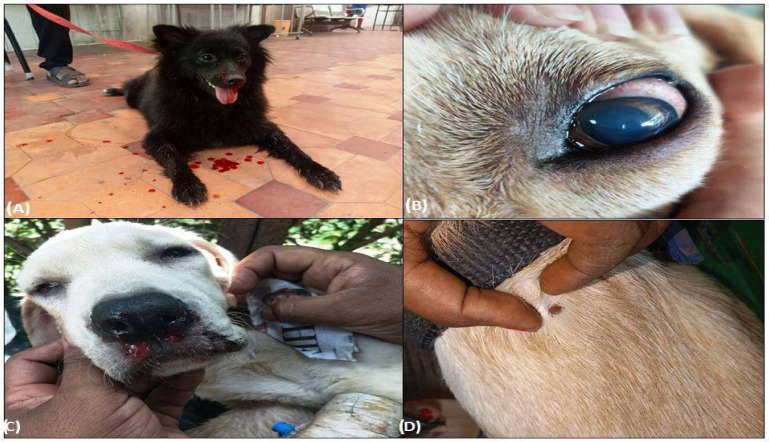
Clinical symptoms of Ehrlichiosis in eastern Indian dogs. (**A**) Epistaxis/Nasal bleeding in affected dogs, (**B**) Corneal opacity in Ehrlichiosis-affected dogs, (**C**) Oedema in affected dogs, (**D**) Presence of ticks on the hair coat of affected dogs.

Typical signs manifested in positively screened dogs were epistaxis (46.4%, Figure 2A), hind limb oedema (16.07%), and distended abdomen suggestive of ascites (26.78%), along with some non-typical signs such as corneal opacity (Figure 2B), uveitis, and icteric sclera. However, most of the dogs showed non-specific symptoms such as fever (75%), lethargy (100%), in-appetence (69.64%), diarrhoea (8.92%), and dullness, etc., making the diagnosis a challenge for the field veterinarians. In one severely affected case, facial oedema (Figure 2C), haemoptysis, and melena were also noticed. Physical examinations of the hair coat in most affected dogs revealed mild to moderate tick infestations (Figure 2D).

### 3.3. Haemato-Biochemical Parameters

A comparative analysis was done for the haematological parameters among the dogs screened positive (*n* = 56) and the dogs found negative for Ehrlichia (*n* = 122), as depicted in Table 3. Statistical analysis through a Student’s t-test showed that there was a significant decrease (*p* ≤ 0.05) in the values of Hb, TLC, PCV, and platelet count, along with neutrophilia and relative leukopenia observed through a differential leucocyte count (DLC) in the ehrlichiosis affected dogs. The serum biochemical alterations observed in the present study are presented in Table 3. A Student’s *t*-test showed a significant (*p* ≤ 0.05) decrease in the albumin levels with a relative increase in the globulin levels and an overall reduction in total protein levels. Other biochemical parameters, including BUN, creatinine, AST, and ALT, observed an increase in the levels indicating liver and kidney anomalies in diseased dogs. 

### 3.4. Urine Examination

A detailed chemical examination of urine samples collected from dogs screened positive for Ehrlichiosis (*n* = 56) recorded proteinuria (69.64%, *n* = 39) and haematuria (10.71%) was a consistent finding. Urine samples from most cases (83.92%, *n* = 47) also showed positive for bile salt and protein levels in affected dogs. There was no change in the pH of the urine samples both in affected and control dogs, which shows an acidic reaction to pH paper.

### 3.5. Ultrasonography

Hepatomegaly (*n* = 7, 12.5%) and a distended gall bladder were observed during ultrasonography, corroborating hepatopathy in Ehrlichiosis-affected dogs. Enlargement of the hepatic artery and vein was noticed through ultrasonography in affected dogs. Dogs suffering from ascites (*n* = 4, 7.14%) showed the presence of hypoechoic to anechoic fluid with floating organs in the abdominal cavity. Splenomegaly was a significant finding, consistently seen in 79% of the affected dogs. Ultrasonography of the kidneys in affected dogs showed mild to moderate hyperechogenic structures suggestive of nephropathy leading to hyperechogenicity, a persistent finding in 25% (*n* = 14) of the positive cases.

### 3.6. Pathology

#### 3.6.1. Gross Pathology

**Figure 3:**
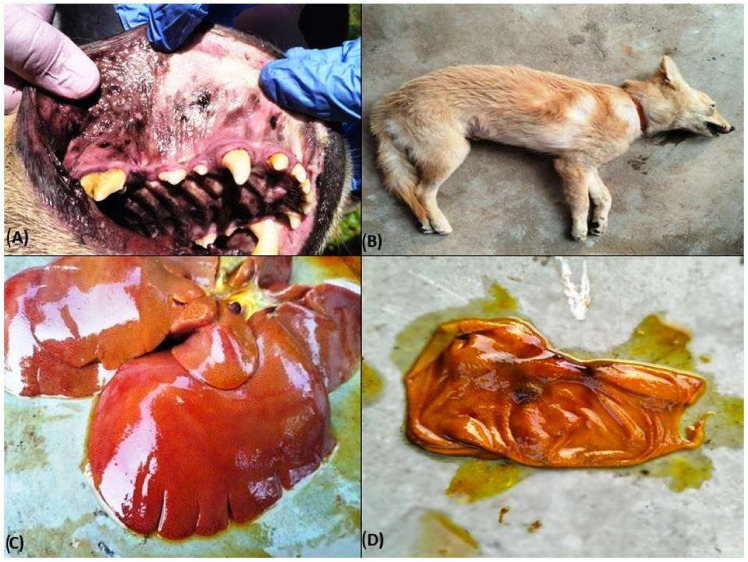
Pathophysiological manifestation in dogs under Ehrlichiosis in eastern India. (**A**) Congested oral mucosae in Ehrlichiosis-affected dogs, (**B**) Hind limb oedema in Ehrlichiosis-affected dogs, (**C**) Enlarged and icteric liver in Ehrlichiosis-affected dogs, (**D**) Haemorrhagic urinary bladder mucosae in Ehrlichiosis affected dogs.

The dogs that died had poor and rough body coats with the presence of ticks on their skin, pale to icteric conjunctiva, petechiae to ecchymotic haemorrhages, and/or congested oral mucosae (Figure 3A) with evidence of nasal bleeding/epistaxis, and presence of scabs in the muzzle. Oedema in the right hind limb was noticed in two carcasses (Figure 3B). Some of the most typical gross lesions included an enlarged spleen and liver. One out of the total three dogs who died also had blood-tinged ascitic fluid in the abdominal cavity. The eyes in affected dogs showed a cloudy appearance, as evidenced during the necropsy. There was hepatomegaly as well as congestion with a distended gall bladder in two cases. At the same time, in one, the liver was icteric with foci of yellow discoloration (Figure 3C). Frothy exudates were found in the upper respiratory tract of the two dogs, with lungs showing typically pneumonic and consolidation. Generalized ecchymotic haemorrhages were present in the liver parenchyma. Kidneys were nodular, contracted with a few necrotic patches on the cortical surfaces, along with firm consistency. Capsules do not easily peel off while removed from the cortex, showing granularity on the cortical surfaces suggestive of nephritis. Most lymph nodes, such as the popliteal, mediastinal, etc., were typically enlarged and congested. Other organs such as the gall bladder and urinary bladder were distended and revealed mild to moderate haemorrhages in the serosa surface (Figure 3D). There were petechiae to ecchymotic haemorrhages in different organs such as the intestine, heart, and lungs, as well as on the intestinal serosa surface. 

#### 3.6.2. Histopathology

**Figure 4:**
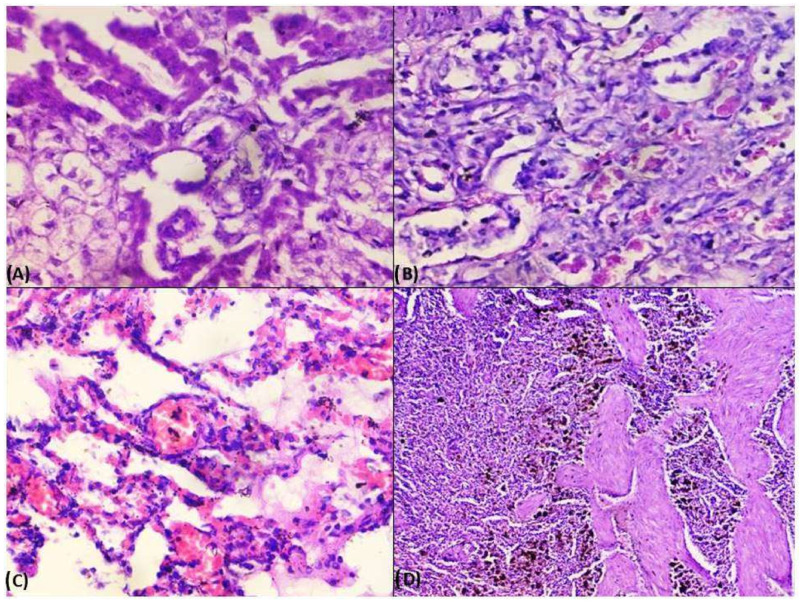
Histological studies in dogs under Ehrlichiosis. (**A**) Photomicrograph showing vacuolar degenerations, fatty changes, necrosis of hepatocytes around the central vein, (**B**) Photomicrograph showing necrosis of renal tubular epithelial cells with desquamation and presence of proteinaceous materials in renal tubules (H&E-40×), (**C**) Photomicrograph showing alveolar degeneration and necrosis along with interstitial congestion and oedema of interstitial spaces (H&E-40×), (**D**) Photomicrograph showing diffuse haemosiderosis in the spleen (H&E-10×).

Microscopic lesions in liver parenchyma were mainly predominated by vacuolar to fatty degenerations, necrosis of hepatocytes around the central vein (Figure 4A), and severe diffuse tri-adenitis in the portal triads, along with centrilobular degeneration and sinusoidal congestion. Multifocal necrosis and degenerations were clearly evident around the central veins of the liver. The liver showed hepatitis with pan-lobular hepatic and fatty degeneration and necrosis of hepatocytes with disorganization of hepatic chords. The kidney also showed periglomerular oedema and focal necrosis with cellular infiltration. The corticomedullary junctions showed plasma cell infiltration. There was also evidence of glomerular necrosis with increased bowman’s space and condensed glomerular tuft. Necrosis of renal tubular epithelial cells with desquamation and the presence of homogenous proteinaceous casts in renal tubules were noticed (Figure 4B). Infiltrations of mononuclear cells in the inter-tubular spaces and fibrotic proliferation were observed in microscopy. There was the presence of a homogenous proteinaceous pink colour renal tubular cast with intertubular congestion. Besides, there were also sub-capsular infiltrations and perivascular infiltrations of inflammatory cells and vasculitis with thickened arterioles with proliferation of the arteriolar wall. Lungs were characterized by interstitial pneumonia with marked fibrosis in the alveolar septum as well as in and around the lung tissue. There was interstitial congestion and thickening of interalveolar septa. There was alveolar degeneration and necrosis along with interstitial congestion and oedema of interstitial spaces (Figure 4C). In the lymph nodes, medullary oedema was prominent, along with plasma cytolysis and follicular hyperplasia. In some cases, we noticed lymphoid follicular depletion as a significant histologic finding in affected dogs. Histological lesions related to diffuse haemosiderosis were detected in splenic sections (Figure 4D).

### 3.7. Identification of Ticks through Scanning Electron Microscopy (SEM)

**Figure 5:**
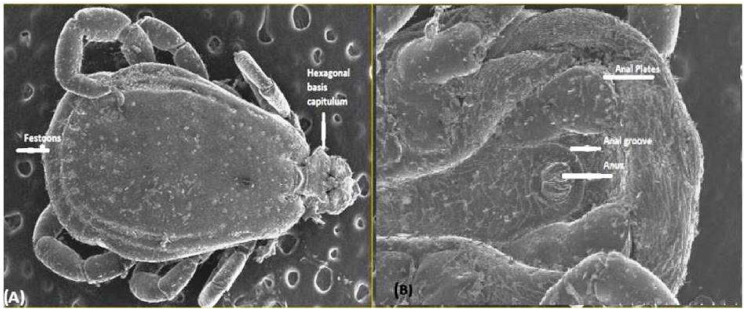
*Rhipicephalus sanguineus* in dog population under Ehrlichiosis. (**A**) Hexagonal basis capitulum and festoons of *R. sanguineus* magnification at 10×, (**B**) The enlarged anal plates, anal groove, and anus of *R. sanguineus* magnification at 100×.

The scanning electron microscopic study of the ticks collected (*n* = 24) from the Ehrlichiosis screened dogs revealed the presence of *Rhipicephalus sanguineus,* determined by the presence of hexagonal basis capitulum (Figure 5A), brevirostrate mouth parts, coxa 1 with two spurs, presence of festoons, anal plates, and anal grooves surrounding anus posteriorly (Figure 5B). Other ticks like *Haemaphysalis* spp. (*n* = 6) also identified by their lateral projection of the second palpal segment, brevirostrate mouthparts, absence of anal plates, and presence of festoons as evidenced through microscopy.

### 3.8. Molecular Confirmation through PCR

**Figure 6:**
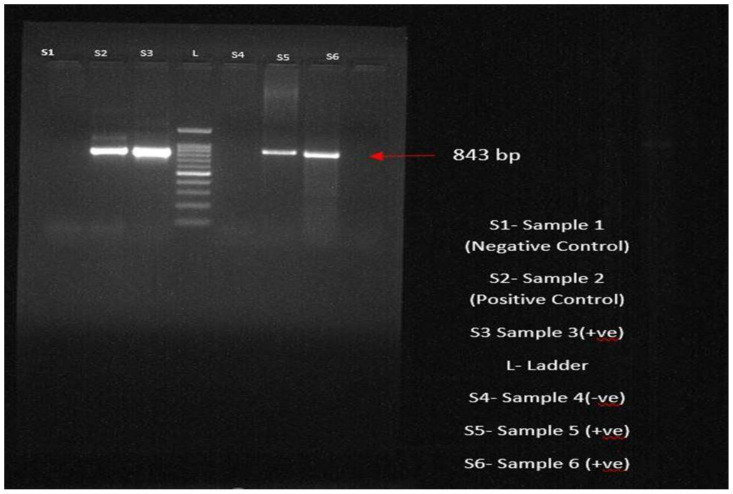
PCR product showing the bands through agarose gel electrophoresis at 843 bp.

All 56 dogs that tested positive through blood and buffy coat smear examinations and clinical examinations had their blood samples sent for PCR examination for molecular conformation of the *Ehrlichia* organism. Among the various diagnostic procedures, the sensitivity and specificity of PCR were highest, revealing 49 positives and showing a prevalence of 87.5%. After DNA extraction following the protocol, the extracted DNA was amplified using *Ehrlichia canis*-specific primers targeting the p28 gene in the thermocycler. The PCR products were analysed using agarose gel electrophoresis; the bands were visualized at 843 bp, indicating the presence of the parasite (Figure 6). A total of 30 numbers of ticks were collected, out of which 24 were identified as *R. sanguineus* and six were identified as *Haemophysalis* spp. 

### 3.9. Phylogenetic Analysis 

**Figure 7:**
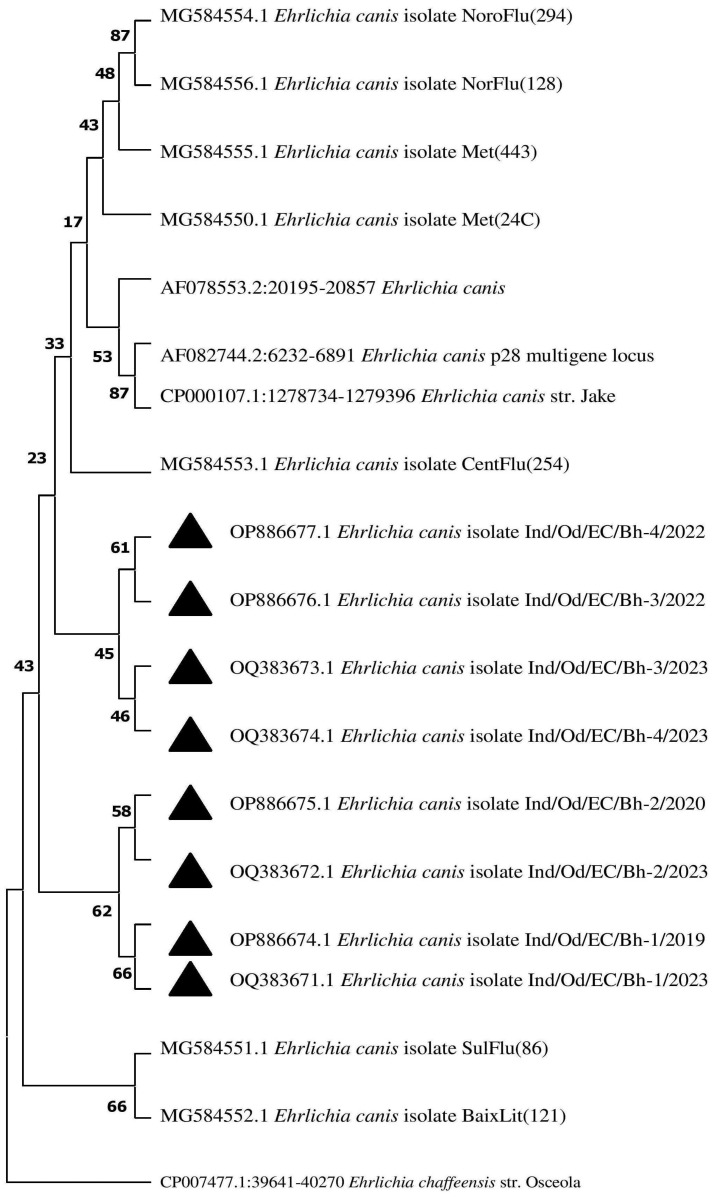
The phylogenetic tree was constructed by using the neighbour-joining (NJ) method with kimura 2- parameters based on nucleotide sequences of the p28 gene of *E. canis.* The black triangle indicates the isolates from the present investigation, whereas *E. chaffeensis* refers to an out-group. The numbers in the node indicate the similarly of the sequences between the compared respective two species.

Using BLAST analysis, the sequences were retrieved from the National Centre for Biotechnology Information (NCBI) database targeting the p28 gene of the parasite. A phylogenetic tree constructed to study the evolutionary relationship revealed that the isolates obtained formed a completely different clade than the existing ones. The isolates of the present investigation revealed a similarity with the isolates derived from the USA with a 100% similarity. The sequence results were sent to the NCBI database, and the following accession numbers were allotted: OQ383671-OQ383674 and OP886674-OP886677, depicted as a black triangle in the Phylogenetic tree (Figure 7). 

### 3.10. Comparative Analysis of the Tests

**Figure 8:**
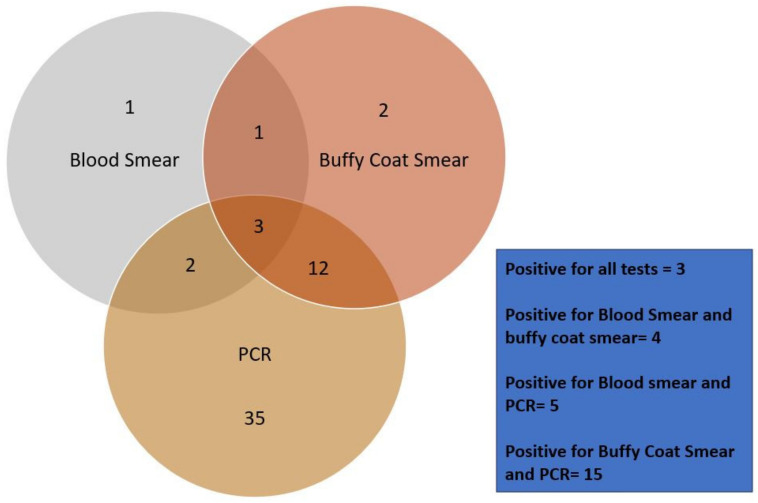
Comparative analysis of various diagnostic methods.

Out of the total 178 dogs under our study, 56 canine blood samples were screened positive using one of the three diagnostic procedures (Figure 8). Out of 56 positively screened samples, only seven samples were screened positive by blood smear examination (12.5%), while eighteen samples with buffy coat smear examination (32.14%), and fifty-two samples from PCR (92.8%) were identified positive, respectively (Chart-1). A relationship had been studied among the tests. Four of the positively screened samples had been positive for both blood smear and buffy coat smear (7.14%). Only five samples were positive by blood smear and PCR (8.92%). Similarly, 15 samples were screened positive by both buffy coat smear and PCR (26.78%), whereas only three were identified as positive with all three tests conducted (5.35%).

## 4. Discussion

The present study recorded a higher prevalence rate of 31.46% Canine Ehrlichiosis in and around Bhubaneswar, Odisha, with a mortality rate of 1.68% and a case fatality rate of 5.35%, which corroborates with the findings of Angkanaporn et al. [8]. The tropical climate at Bhubaneswar mostly favours vector development as well as transmission among the susceptible population [16], attributing a higher prevalence. The low sensitivity of detecting the morulae through blood smear examination, as evident in the present study, might be associated with low parasitaemia and variability in the stage of infection in the host body [13]. Guedes et al. [17] mentioned that morula is visible only in the acute phase of the disease during higher parasitaemia. This statement justifies the lower positivity rates obtained during our study by the blood and buffy coat smear examinations. Although most of the morula stages are visible in the monocyte, hence the name Canine Monocytic Ehrlichiosis (CME), according to our findings, morula stages were also evident in neutrophils and lymphocytes. Similar findings were reported by Selim et al. [2]. 

Although there is no strict age-based predilection, the younger animals are more susceptible due to their less developed immune responses and a sudden decrease in the maternal antibodies after three months of age [18]. Our findings of higher occurrences in males could be justified because males are more prone to tick infestation due to their aggressive behavioural attributes, as stated by Okubanjo et al. [19]. Pet owners mostly prefer to keep male dogs, which is attributed to the higher prevalence of Ehrlichiosis in the present study [12]. The reason why the heavier breeds are more prone to the occurrence of Ehrlichiosis could be endorsed by the fact that the larger breed dogs have more surface area and good body conditions. They also have less immunity than do the local non-descript breeds, and they frequently suffer from tick infestation and eventually become prone to Ehrlichiosis. Out of other heavy breeds, the German Shepherd is the most susceptible due to its inherent breed inability for blast formation and its leucocyte migration inhibition factor, as stated by Bharadwaj et al. [20], Himalini et al. [21], and Bhadesiya and Rawal [22]. 

Higher occurrences of Ehrlichiosis in the Labrador breed in the present study are attributed to heavy hair coats, which often go unnoticed by most pet owners, thus making them more susceptible to easy stay and transmission of the disease [21]. The heavyweight of any animal makes them more vulnerable to parasitic and mainly tick infestations due to their larger body area, which further acts as a good source of nutrition for the parasite. Our observations about housing conditions were in agreement with the findings reported by Bhadesia and Raval [22], attributed to maximum tick exposure in an outside environment. *Riphicephalus* ticks are more common in urban areas, which correlates with our findings [23,24]. These observations could be justified since the tick population thrives best during hot and humid conditions, favouring tick development by increasing the chances of laying and hatching eggs and nymphal moulting [25]. The symptoms observed in our study were in agreement with Tuna et al. [26] and Haritha et al. [27]. Symptoms such as fever are consistently found in the majority of dogs because of the increased production of interleukin-1 (IL-1) by antigen-presenting cells and B-cells, or exogenous pyrogen products of the rickettsia organism, as stated by Rungsipipat et al. [28].

Most of the affected animals in our study showed a normocytic normochromic type of anaemia attributed to immune-mediated disorders and suppression of bone marrow, as reported by Thongsahuas et al. [29] and Siarkou et al. [30]. Anaemia observed in the acute stage is credited to increased platelet consumption, vasculitis, and splenic sequestration; however, in the chronic stage, it occurs mainly due to bone marrow hypoplasia leading to pancytopenia, as justified by Angkanaporn et al. [8]. The increased value of ALT and AST indicates hepatic involvement in the pathogenesis of Canine Ehrlichiosis. Further, the elevated levels of BUN and creatinine justified the renal apathy during the disease. Hypergammaglobulinemia is a consistent feature of Canine Ehrlichiosis. However, the levels of globulin do not correlate with the levels of antibodies of *E. canis*, indicating that most of the globulins responsible for the total increase in gamma globulin were not antibodies to *E. canis* [31]. Serum biochemical alterations related to increased BUN and creatinine levels corroborated with nephropathy in the present study.

It was found that *Rhipicephalus sanguineus* (Brown dog tick) was an important vector responsible for the transmission of this disease in this area, morphologically identified through a scanning electron microscopic (SEM) study. Present findings corroborate earlier researchers such as Kottadamane et al. [32] and Neave et al. [33]. Bhubaneswar climatic conditions are more or less similar to tropical climates, with hot and humid conditions favoring tick multiplication and disease transmission among susceptible dogs [8].

Hepatomegaly, gall bladder distension, and the presence of clear/sludge bile were observed as a typical finding because of the anorectic condition of the affected dogs in canine ehrlichiosis (Kumar et al. 2012). Ultra-sonographic findings recorded in the present study corroborated earlier reports of Angkanaporn et al. [8]. The necropsy lesions were consistent with the findings reported by Behera et al. (2012). Splenomegaly is a characteristic finding similar to our observations, owing to the fact that the spleen acts as a significant organ for harbouring Ehrlichia-infected macrophages, which eventually leads to the destruction of platelets [34]. Similar findings in histopathological analysis were also reported by Mylonakis et al. [35] and Waner et al. [10]. 

The PCR has been proven to be the most accurate, quick, and convenient method of diagnosis with the highest specificity and sensitivity as compared to the other approaches used [33]. Nakaghi et al. [13] stated the high specificity of PCR can be accounted for by its ability to detect Ehrlichia DNA with ricketsaemia as low as one infected monocyte in 10^36^ cells [36,37,38]. 

## 5. Conclusions

Tick-borne diseases in dogs impede the health of dogs and cause detrimental effects on their overall performance and immunity. Early detection of the disease with suitable veterinary care may limit unnecessary veterinary expenses along with increasing the survivability of the dogs. This molecular report, recorded as the first-ever detailed investigative approach on this significant emerging disease with relatively higher prevalence in Bhubaneswar of Eastern India, needs the proper attention of all the stakeholders to mitigate further spread and to enrich the scientific literature about the disease in this corner of the globe. Apart from the conventional screening through blood smear examination, a molecular approach like PCR should be advocated for, owing to its sensitivity and specificity. 

## Figures and Tables

**Table 1:** Temperature time Protocol in the Proflex PCR system (Invitrogen, Waltham, MA, USA).

Events	Temperature	Time	Cycles
Initial Denaturation	95 °C	5 min	1 cycle
Denaturation, Annealing, and Extension	95 °C	30 s	35 cycles
	55 °C	1 min	
	72 °C	2 min	
Extension	72 °C	5 min	1 cycle

**Table 2:** Detailed epidemiological risk factors in canine ehrlichiosis.

Variable		N	Positive	Prevalence (%)	OR	95% CI	Chi Square	*p* Value
Gender	Male	115	38	33.04	*	-	>0.9	
	Female	57	18	31.57	1.06	0.50–2.24		0.9
Age (years)	<1	51	23	45.09	*	-	0.045	
	1 to 2	36	12	33.33	0.37	0.14–0.89		0.030
	>2	79	21	26.58	0.39	0.17–0.87		0.022
Ticks	Absent	38	12	31.57	*	-	>0.9	
	Present	140	44	31.42	0.88	0.38–2.06		0.8
Housing	Kaccha House with Field	51	16	31.37	*	-	>0.9	
	Pukka house with close confinement	22	7	31.81	0.89	0.27–2.83		0.9
	Pukka house with access to open field	105	33	31.42	1	0.47–2.19		>0.9
Body weight (kg)	<10	16	5	31.25	*	-	>0.9	
	10 to 20	29	9	31.03	1.08	0.28–4.51		>0.9
	>20	133	42	31.57	0.88	0.28–3.11		0.8
Season	Rainy	54	17	31.48	*	-	>0.9	
	Summer	98	31	31.63	1.16	0.54–2.54		0.7
	Winter	26	8	30.76	1.03	0.34–2.98		>0.9
Breed	G.S	44	14	31.81	*	-	>0.9	
	Labrador	39	12	30.76	1	0.38–2.67		>0.9
	Golden Retriever	28	9	32.14	0.97	0.33–2.78		>0.9
	Alsatian	25	8	32	0.93	0.30–2.79		>0.9
	Doberman	19	6	31.57	1.11	0.31–3.74		0.9
	Spitz	13	4	30.76	0.8	0.18–3.13		0.8
	Beagle	10	3	30	0.99	0.18–4.42		>0.9

* indicates the reference group with which other groups of that criteria are compared.

**Table 3:** Serum biochemical alterations in dogs affected with Ehrlichiosis.

Parameters	Ehrlichiosis Negative (N = 122)	Ehrlichiosis Positive (N = 56)
Hb (g/dL)	14.07 ± 0.30 ^a^	6.33 ± 0.22 ^b^
TEC (×10^6^ µL)	7.43 ± 1.04 ^a^	3.47 ± 0.19 ^b^
PCV (%)	44.10 ± 0.88 ^a^	19.33 ± 0.74 ^b^
TLC (×10^3^ µL)	10.87 ± 0.63 ^a^	9.40 ± 0.13 ^b^
PLT	532.20 ± 38.68 ^a^	74.40 ± 4.31 ^b^
MCV (fl)	59.81 ± 1.24 ^a^	64.14 ± 0.38 ^b^
MCH (Pg)	22.17 ± 0.13 ^a^	23.07 ± 0.08 ^b^
MCHC (%)	33.46 ± 0.38 ^a^	38.23 ± 0.21 ^b^
N (%)	57.20 ± 0.38 ^a^	78.37 ± 0.83 ^b^
L (%)	36.70 ± 0.35 ^a^	16.50 ± 0.79 ^b^
E (%)	4.47 ± 0.26 ^a^	1.53 ± 0.93 ^b^
M (%)	1.63 ± 0.89 ^a^	3.60 ± 0.13 ^b^

Data are presented as mean ± SE. Mean values with different superscripts differ significantly (*p* < 0.01).

## Data Availability

All data included in the article are referenced in this article. Any individual data shall be provided by the corresponding authors upon request.
